# Scurvy

**Published:** 1889-10-12

**Authors:** 


### THE PRACTITIONER'S OUTLOOK AND RETROSPECT.

### MEDICINE.

### SCURVY.

It is mentioned in one of the medical journals that Dr. Talysin, having under his care two cases of syphilis complicated by scurvy, and finding no mention in literature of the best treatment of the two diseases when occurring simultaneously, resolved to treat the syphilis alone. Mercury was given, which produced a considerable degree of cachexia, but cured the syphilis. Then the scurvy was treated. Now, the complication of scurvy with syphilis is a very common condition, especially in the East, where land scurvy is endemic, and the frequent prevalence of both maladies in the same patient is prominently noticed by authors on the diseases of India. We do not feel by any means sure that the best plan in such a case is to treat the syphilis first and the scurvy afterwards. There is no legitimate reason why anti-syphilitic remedies and anti-scorbutics should not be given at the same time. If any reason should present, we would certainly be disposed to treat the scurvy first. When a scurvy taint exists in the blood, various affections, both medical and surgical, often defy all treatment until anti-scorbutics are administered; and syphilis is not often an exception, as it appears to have been in Dr. Talysin's case. And it may be serviceably recollected that there is such a condition as *latent scurvy*—that is, the scurvy taint may exist in the blood, without the development of the spongy gums and indurations marking the patent disease. For instance, the non-healing of an ulcer or sore, or the failure of the specific action of quinine in malarious fevers, may be due to a scurvy taint. It is therefore well, in countries where scurvy is endemic, or when persons have been placed in positions where fresh vegetables were scarce, to administer anti-scorbutics when ordinary diseases are not amenable to ordinary remedies, even although no visible signs of scurvy present. Anti-scorbutics will not in any case do harm, and may be productive of great benefit.

